# Views of people who have given birth on the environmental and occupational exposure risks of nitrous oxide for labour analgesia: an interview‐based qualitative study

**DOI:** 10.1111/anae.16687

**Published:** 2025-07-29

**Authors:** Laura Elgie, Tom Salih, S. Ramani Moonesinghe

**Affiliations:** ^1^ Department of Targeted Intervention University College London London UK; ^2^ Department of Anaesthesia and Perioperative Medicine University College London Hospitals London UK

**Keywords:** Entonox, environment, nitrous oxide, occupational exposure, parturition

## Abstract

**Introduction:**

A 50:50 nitrous oxide/oxygen mix is commonly used for labour analgesia in the UK; however, nitrous oxide is associated with comparatively high environmental impact and potential exposure risks to staff if appropriate measures are not taken. Reduction of the impact of nitrous oxide in line with net zero emissions targets would require widespread changes in the way nitrous oxide is administered or in the availability of alternative options for labour analgesia. The aims of this qualitative study were to understand nitrous oxide use from the perspective of the parturient in the context of concerns regarding the environment and occupational exposure, and to provide evidence to guide the development of patient‐centred nitrous oxide mitigation strategies.

**Methods:**

We conducted semi‐structured interviews with participants who had used nitrous oxide for labour analgesia. Participants were recruited purposively. Data were analysed using reflexive thematic analysis.

**Results:**

We conducted 12 interviews and identified three themes: nitrous oxide is the default for labour analgesia in the UK; mitigating nitrous oxide harm depends on personal priorities; and institutions have a responsibility to mitigate nitrous oxide harm. Participants viewed nitrous oxide as a safe option, readily available in the context of limited choices for labour analgesia. They would be interested to know about the environmental risk of nitrous oxide, though this would not necessarily influence their choice to use it. They were less interested in its occupational risks and considered institutions, rather than individuals in labour, to have a responsibility to mitigate these. Though they felt that investment in reducing NHS emissions is important, participants also felt there may be other more pressing financial priorities.

**Discussion:**

This study provides evidence to support the development of strategies to reduce nitrous oxide emissions, which consider the views of people who have used nitrous oxide for labour analgesia.

## Plain Language Summary

In the UK, many people use a gas called nitrous oxide mixed with oxygen to help with pain during childbirth. But nitrous oxide can harm the environment and may not be safe for hospital staff if not handled properly. To help protect the planet and reach ‘net zero’ (which means not adding more pollution than we take away), hospitals might need to change how they use this gas or find other ways to help with pain during labour. This study wanted to understand what people who used nitrous oxide during childbirth think, especially about how it affects the environment and hospital staff. We talked to 12 people who used nitrous oxide during labour. We asked them open‐ended questions to hear their thoughts. Then we looked for patterns in what they said. We found three main ideas: nitrous oxide is the usual choice for pain relief during labour in the UK; people care about the environment, but what matters most depends on their personal situation; and people think hospitals, not mothers, should take care of making nitrous oxide safer for the environment and staff. Most people felt nitrous oxide was a safe and easy option, especially when they didn’t have many choices. They were curious to learn more about how it harms the environment but said this might not stop them from using it. They weren’t too worried about the risks to staff and thought hospitals should be the ones to fix that. While they supported helping the environment, they also thought the NHS might have more urgent needs for its money. This study gives helpful ideas for creating plans to reduce nitrous oxide pollution that also respect the needs and opinions of people who use it during childbirth.

## Introduction

A 50:50, nitrous oxide/oxygen mix (e.g. Entonox^®^, commonly referred to as ‘gas and air’ in the UK) is the most common form of labour analgesia in the UK [1]. It has been used for over 50 years [2] and is required to be available in all childbirth settings [3]. It is used widely in several other countries including Australia, Canada and parts of Scandinavia [4, 5]. However, nitrous oxide is a potent greenhouse gas responsible for up to 75% of the total anaesthetic gas footprint [6]. Over 60% of its use takes place in maternity units [7] and it has the greatest global warming potential of labour analgesics [8]. In addition, nitrous oxide is associated with potential toxicity to staff who are exposed to high levels for long periods of time. Systemic effects related to the inactivation of vitamin B12 include reproductive toxicity and neurological effects [9], although there is no evidence for toxicity within occupational limits [10]. In recent years, nitrous oxide has been temporarily withdrawn from several UK maternity units for exceeding occupational limits [11].

There are several potential strategies to reduce nitrous oxide emissions in line with NHS Net Zero targets [12, 13], addressing both pure nitrous oxide and nitrous oxide/oxygen mix. This includes improving the efficiency of nitrous oxide delivery [14]; reduction in clinical use; and introducing waste gas treatment technology [15]. Up to 100% of pure nitrous oxide is wasted. This is most commonly via manifold system leaks, poor stock management and inadequate security [14]. Early evidence from piped nitrous oxide/oxygen mix waste mitigation projects suggests it is wasted for the same reasons [7]. Considerable emissions reductions can be made without significant change in clinical practice at the point of use. However, reduction in the clinical use of nitrous oxide for labour analgesia requires alternative analgesia to be considered acceptable and made available to people in labour [12]. Waste gas treatment technologies require further validation for effectiveness before they can be recommended universally [16] and, since they rely on users exhaling into a mask or mouthpiece, will require appropriate technique by users.

Maternal satisfaction with nitrous oxide is high despite limited evidence for its analgesic effectiveness [4, 17]. Although patient‐focused studies have reported the usefulness of nitrous oxide for labour analgesia [18], they have not included environmental considerations. Climate change is a topic of concern for health users, but only one quarter of people surveyed by the Health Foundation believed that the NHS is contributory [19]. There is limited information about how implementation of measures to mitigate nitrous oxide emissions will impact people in labour and whether awareness of environmental or occupational exposure concerns is associated with decision‐making in labour. It has been suggested that the environmental impact of nitrous oxide should be discussed with pregnant people, because it is a risk they may want to factor into their decision‐making when considering their choice of labour analgesia [20]. Person‐centred care necessitates consideration of these issues [21]. Removal of analgesic options can perpetuate inequity in care, whilst climate anxiety can cause significant distress [22, 23].

The aim of this study was to explore the views of people who have used nitrous oxide for labour analgesia to provide evidence for the development of patient‐centred nitrous oxide emission and occupational exposure reduction strategies. We conducted a qualitative study with two research objectives. First, to discover the views of people who have used nitrous oxide for labour analgesia on its environmental and occupational risks. Second, to explore how strategies for the reduction of harm related to nitrous oxide could be implemented in a way that is most acceptable to people giving birth.

## Methods

This study was approved by the UCL Research Ethics Committee and is reported according to the Standards for Reporting Qualitative Research checklist [24].

Potential participants were eligible for inclusion if they had given birth and used nitrous oxide for labour analgesia within the previous year and were recruited through advertisements placed in children's groups and parents' social media networks (online Supporting Information Appendix S1). Potential participants were sent a plain language information sheet (online Supporting Information Appendix S2) and consent form. Participants were purposively selected from advert respondents with the aim of capturing rich and varied data, for example by including a participant who had used nitrous oxide for home birth. Interviewees were given a £15 voucher (US$21, €18) to thank them for their participation.

Data were collected via interviews over a 3‐month period (July–September 2023). Ten interviews were conducted on Microsoft Teams (Microsoft Corporation, Redmond, WA, USA), one by telephone and one face‐to‐face. All interviews were recorded and transcribed using Microsoft Teams, with transcripts corrected against the recordings by the same author who had conducted the interviews (LE). Transcripts of the recordings were pseudonymised and stored securely according to local data protection guidelines. Interviews were semi‐structured using an interview guide that included prompts for more depth where appropriate (online Supporting Information Appendix S3). The interview questions were developed by the authors based on the study objectives in the context of nitrous oxide impact and mitigation strategies identified in our literature search. The opening questions helped us establish rapport with our study participants and explore their individual experience of using nitrous oxide for labour analgesia. We separated topics related to awareness of potential impact and asked these questions before presenting participants with information about environmental and occupational effects. The interview guide was modified after two trial interviews, following which we adapted some of our language and prompts. For example, our trial interviewees spoke about ‘gas and air’ rather than ‘nitrous oxide’ and ‘Entonox^®^’, so we used the former term in subsequent interviews.

We chose our sample size to achieve sufficient information power for our study objectives. According to the model by Malterud et al., sample size is determined by the amount of information it contains so “*the more information that the sample holds, relevant for the actual study, the lower the number of participants that is needed*” [25]. The model also recommends that “*an approximation of sample size is necessary for planning, while the adequacy of the final sample must be continuously evaluated during the research process*” [25]. We planned an approximate sample size of 10 participants based on a similar study of midwives [26] and evaluated the information power attained during the research process continuously. Features of our study that were designed to produce high information power were: the narrow study aims; seeking to identify participants' views on specific questions; the dense sample specificity achieved through purposive sampling; and the strong quality of dialogue, with participants expressing themselves articulately and developing good rapport with the interviewer during the interviews. We considered we had achieved adequate information power to develop new knowledge and meet our study aims after 12 interviews.

We did not align our work with an established theory and sought to develop an understanding of participants' views based on an inductive approach to the data. We used reflexive thematic analysis to best answer questions relating to ‘why’ nitrous oxide analgesia use is widespread and ‘how’ its environmental and occupational effects can be addressed without compromising labour analgesia options for people giving birth. Reflexive thematic analysis is a method of identifying and developing patterns of meaning within qualitative data, which foregrounds researcher subjectivity [27, 28]. This is particularly relevant as we, the authors, are insiders in the anaesthetic field, and cannot separate this from our interpretations and analysis. Themes are analytic outputs, created as “*interpretive stories about the data, produced at the intersection of the researcher's theoretical assumptions, their analytic resources and skill and the data themselves*” [27]. We are not aware of other research on the opinions of people who have given birth on the risks of nitrous oxide and how to mitigate these, so we considered it important to adopt an approach that gave voice to our participants and was directed by the content of our data (inductive) rather than by existing concepts (deductive) [29].

Pseudonymised interview transcripts were analysed in NVivo 12 (QSR International, Burlington. MA, USA). A single transcript was coded collaboratively by two authors (LE and TS) through discussion and reflection of the authors ideas and assumptions [30]. This collaboration was to enhance reflexive engagement with the data rather than achieve perceived accurate coding [30]. One author (LE) coded the remaining transcripts; most coding was semantic, capturing the explicit meaning of what interviewees were saying, and some was latent, bringing together concepts that underpinned interviewees' responses. We developed themes and subthemes based on broader patterns of meaning in the codes. This was an iterative process involving going back and forth between codes, merging codes which overlapped and ensuring themes captured shared meanings. Two authors (LE and TS) held multiple reflexive discussions during this phase. Data analysis continued during the writing of this study by going back to codes, clarifying theme boundaries and placing the study results in the context of existing literature. When quoting from participants, we re‐read the quotes within the original transcripts to ensure the quote was representative of the participants' views.

With regards to reflexivity, the analysis presented here stems from our interest in environmental sustainability in healthcare and belief that people giving birth should be able to access nitrous oxide for analgesia. We are involved in the development of technology to reduce nitrous oxide emissions and approached questions on nitrous oxide mitigation strategies in this context. We have previously discussed that addressing the environmental and occupational concerns of nitrous oxide are closely linked [31]. One author (LE) also approached this study as someone who found nitrous oxide effective for labour analgesia.

## Results

The interviews lasted 19–35 min. Seven participants had given birth once and five had given birth twice. In the latter, the interviews focused on their most recent delivery, though they had all also used nitrous oxide for labour analgesia for their first delivery. Ten participants delivered in an obstetrician‐led delivery suite, one at home, and one in a midwife‐led birth centre. All participants used nitrous oxide for labour analgesia and three recall using it postnatally during suturing. Nitrous oxide was the only form of pharmacological analgesia used by nine participants, though three of those nine did ask for an epidural but did not receive one. Three other participants had an epidural and stopped using nitrous oxide once that was sited. Eight participants gave birth by spontaneous vaginal delivery, one by instrumental delivery and three by urgent caesarean delivery.

Of the 12 interviewees, one was aware of the harmful environmental effects of nitrous oxide before participating in this study and two were aware, though had limited understanding, of its potential to cause occupational harm. Eleven interviewees thought that the environmental effects of nitrous oxide and other labour analgesics should be discussed antenatally. There was a mix of opinion about whether the occupational exposure effects of nitrous oxide should be discussed antenatally; interviewees acknowledged that this could be a difficult conversation to have with a midwife. Environmental and occupational concerns had not factored into any of the participants' previous use of nitrous oxide for labour analgesia. A summary of the themes and subthemes is presented in Table 1.

**Table 1:** Summary of themes and subthemes.

Theme	Subtheme	Illustrative quotation
Nitrous oxide analgesia is the default for labour analgesia	Nitrous oxide analgesia feels like the safe option	“*The other painkillers like the next stage, like the stronger ones…I wouldn't have felt comfortable taking those for my baby*” (Participant 2)
	Nitrous oxide analgesia is readily available in multiple settings; it's just there	“*Someone just handed it to me when I asked for it*” (Participant 10)
	There are not many other options	“…*because there's such limited [pain relief] options available to you really*” (Participant 4)
	Midwives' influence	“*…I was just relying on what the midwife did and what she gave me*” (Participant 2)
	Expectation that nitrous oxide analgesia will be available	“*Knowing there's something that you can take for a bit of immediate release, that's what I just think feels really scary to me if that wasn't there*” (Participant 3)
Mitigating nitrous oxide harm depends on personal priorities	Timely information	“*I'm not sure if it [knowing about environmental effects of nitrous oxide analgesia] would have affected my decision, but I would have liked to at least had time to think about it and digest it*” (Participant 7)
	Pain management over environment	“*I do care about the environment and things like that, but for me, I think I do my bit elsewhere…so I wouldn't really care about the environmental effects [of nitrous oxide analgesia]*” (Participant 5)
	Maintaining the benefits of nitrous oxide analgesia	“*I like that it's something that you can self‐administer and it helps you control your journey*” (Participant 3)
Institutions have a responsibility to mitigate nitrous oxide harm	Responsibility to protect midwives from occupational risk	“*I feel like this is the hospital's responsibility*” (Participant 1)
	Responsibility to people giving birth	“…*it's slightly unfair to take that [nitrous oxide analgesia] away from women*” (Participant 10)
	Responsibility to reduce nitrous oxide impact in the context of other emissions	“*Every single thing, everything could be reusable and sanitised and cleaned properly…And so, what is the impact of all of those other things in terms of carbon dioxide or whatever the measures are? And if this [nitrous oxide analgesia] is top of the list, then yeah, it should definitely be prioritised. But I think there's lots of other things that contribute that probably also need to be prioritised*” (Participant 8)
	Responsibility to balance nitrous oxide mitigation investment against other priorities	“*I would be quite happy for the NHS to, you know, spend a fair amount on it [nitrous oxide mitigation] providing everything else worked. If you know what I mean…like is that where the money should be spent if we can't even get the right number of hospital beds we need for the patients that we've got?*” (Participant 12)

### Theme: nitrous oxide is the default for labour analgesia

Interviewees saw nitrous oxide for labour analgesia as the safe option, to the extent that one participant described it as something “*that's available that wasn't seen as a drug in inverted commas*” (Participant 11). Nitrous oxide was seen as “*really good pain relief…it's not so harsh like the other ones*” (Participant 5) and a safe option for mums and babies, where “*it just sounded like the best option for me not to be pumped with lots of chemicals having an effect on the baby*” (Participant 2). Interviewees gave the impression that nitrous oxide was being promoted to them over other forms of labour analgesia at private antenatal classes where “*there's a bit of a steer away from epidurals…so gas and air was…really the only option*” (Participant 4).

Participants felt that nitrous oxide is readily available at short notice in multiple settings, unlike other forms of pharmacological labour analgesia which can require more time to arrange and need to be administered in a monitored environment. Participants described using it during homebirth “*the midwife came to our house…and she had two cannisters of gas and air*” (Participant 2); at the maternity assessment unit “*on the wheelchair*” (Participant 8); and in an ambulance “*they gave me gas and air straight away*” (Participant 5).

There are “*limited [labour analgesia] options available*” (Participant 4). One interviewee described that “*there just seemed nothing else available [other than nitrous oxide]*” (Participant 10). Even when a parturient explicitly asked for other forms of labour analgesia, in some cases it was the only analgesia used: “*I wanted to have epidural if possible…but because the doctor was busy, I didn't have it, they could only offer me the gas*” (Participant 1).

Midwives influenced what labour analgesia interviewees received and when. Given the study population, all interviewees were initially offered (or specifically asked for) nitrous oxide for labour analgesia. One participant progressed from nitrous oxide to an epidural, on the advice of her midwives, even though “*I couldn't feel any of the contractions, the only reason I moved on to the epidural is because…I'd been on it a while. They [the midwives] said if you take the epidural, you just go to sleep…*” (Participant 5). Another participant continued to use nitrous oxide on the advice of her midwife, even though it “*[didn't] feel effective, but I did what I was told*” (Participant 1). Interviewees used particular language when describing aspects of their labour such as “*you're not really present enough [during labour]*” (Participant 11) and “*you're quite out of control*” (Participant 3), which made us appreciate their potential vulnerability during labour and how significant the role and influence of the midwife is in their experience.

There was a broad expectation that nitrous oxide would be available for labour analgesia. In the case of the multiparous participants, their previous experience of labour analgesia influenced their choice and expectations of subsequent labour analgesia: “*I chose to do that like that [with nitrous oxide] because I'd liked it with my first baby*” (Participant 2). The interviews were conducted in the months following the withdrawal of nitrous oxide from several UK maternity units due to occupational exposure concerns. Interviewees described expecting to receive labour analgesia quickly: “*…if they didn't have it, then I would expect another form of pain relief quite promptly…*” (Participant 5). The prospect of nitrous oxide not being available “*feels really scary to me*” (Participant 3) particularly for people planning to deliver in a midwife‐led unit, because “*if that wasn't there, then what would be the option for me?*” (Participant 8). One French participant explained that nitrous oxide is not used for labour analgesia in France and, for her, it “*…was just like a bonus…it wasn't something I was thinking about before being pregnant or before being pregnant in the UK …*” (Participant 9).

### Theme: mitigating nitrous oxide harm depends on personal priorities

Receiving timely information on the environmental impact of labour analgesia appeared important to our participants. This can be at “a *time where you're doing…birth planning and you have space to…weigh up the information*” (Participant 6). Participants were concerned about pregnant people feeling guilty for choosing to use nitrous oxide for labour analgesia, so the information could be “*at least signposted to…but perhaps not enforced at time when…women are feeling quite vulnerable*” (Participant 7). Though there was broad consensus that “*environmental concerns are important to discuss…for many women they will be something that they are very, very concerned about*” (Participant 7). One participant disagreed because “*…climate change, it's a sweeping statement…it's branded around so much these days…I wonder if it [nitrous oxide] is going to make much of a difference to the environment at all, so I don't think it's fair to discuss it [with pregnant people]*” (Participant 10).

Participants felt that an individual's pain management should be prioritised over the environmental effects of nitrous oxide. Knowing about these effects may not be “*enough to convince me to get an epidural*” (Participant 4). Some participants, however, felt that knowledge of the environmental impact of nitrous oxide may affect the point at which they start to use it or the duration of time which they use it for, for example “*if you're early on in your labour, [you might think] do I really need it at this point?*” (Participant 3).

Participants thought that mitigation strategies should maintain the benefits of nitrous oxide for labour analgesia in a safe way. Participants wanted to know that any new technology offered to them “*was a bit tried and tested*” (Participant 5) with “*enough evidence to share that it was safe*” (Participant 8). All participants would be happy to use nitrous oxide mitigation strategies “*if the effect [of nitrous oxide] is still the same*” (Participant 2). This includes the non‐analgesic benefits of nitrous oxide, such as having “*something to focus on*” (Participant 4), being “*something that you can self‐administer*” (Participant 3) and the ability to remain mobile whilst using it. For one participant, for example, “*if the alternative [mitigation strategy] meant that I couldn't be walking around, or I couldn't be in and out of the pool and I had to be in a fixed position in order to use the alternative [mitigation] technology…I would probably choose not to use it*” (Participant 12).

### Theme: institutions have a responsibility to mitigate nitrous oxide harm

Participants felt that the responsibility to protect midwives from occupational risk is not “*the concern of the labouring woman*”, *but* “*…is the concern of the institution they work for*” (Participant 12). Occupational risk “*should probably be thought of at a…more senior systemic level within the NHS…rather than putting the responsibility on women who are in labour…*” (Participant 6). Participants' responses regarding whether pregnant people should be informed of the occupational exposure risks of nitrous oxide were mixed, with many respondents imagining that this would be a “*difficult conversation to have [with your midwife]*” (Participant 10) and might make them “*feel guilty about my choice of pain relief because of the effects on staff that were…out of my control*” (Participant 4).

Participants felt that Trusts have a responsibility to people giving birth, with a sense that “*money isn't invested as well as it could be to look after women and babies*” (Participant 6). Participants described concern that “*women are being punished for [nitrous oxide emissions]*” (Participant 10) and “*…just to target childbirth and women's health, again feels a little bit personal…*” (Participant 3). Related to the limited analgesia options discussed above, a complementary way of approaching nitrous oxide mitigation is supporting research into alternative labour analgesia, to have “*some different pain relief options*” (Participant 4).

Participants felt that Trusts have a responsibility to reduce nitrous oxide emissions in the context of NHS emissions as a whole, with participants interested to know “*Is it [nitrous oxide for labour analgesia] the biggest contributor or is it having the biggest negative impact on the environment out of everything that the healthcare profession provides?*” (Participant 12). Though the cumulative effect of nitrous oxide use was recognised as it has “*been used for a long time…there are a lot of births happening at a lot of time*” (Participant 11), others felt that “*there are other situations which we could reduce emissions apart from giving birth*” (Participant 10).

Participants felt that Trusts are responsible for balancing nitrous oxide mitigation investment against other financial priorities, such as “*the staff…I think that should be foremost what gets sorted and then you can look at the environmental stuff*” (Participant 5). At the same time, “*ensuring that the NHS is sort of sustainable, minimising its environmental impact like in the long term, that is what's best for everyone*” (Participant 7). Occupational exposure risks of nitrous oxide should be factored in when discussing investment into nitrous oxide emission mitigation technologies *because* “*when it [the environmental risk of nitrous oxide] is coupled with risk to staff as well…the two together feel like quite [an] immediate concern*” (Participant 3).

We asked participants how much money they thought was reasonable to spend on nitrous oxide mitigation in relation to the cost of perinatal care. This was universally difficult to answer with responses such as “*it's hard to tell, to be honest, it's really hard*” (Participant 9) and, despite reflecting between interviews on how to probe this from a different angle, we did not collect rich data in this area.

## Discussion

**Figure 1:**
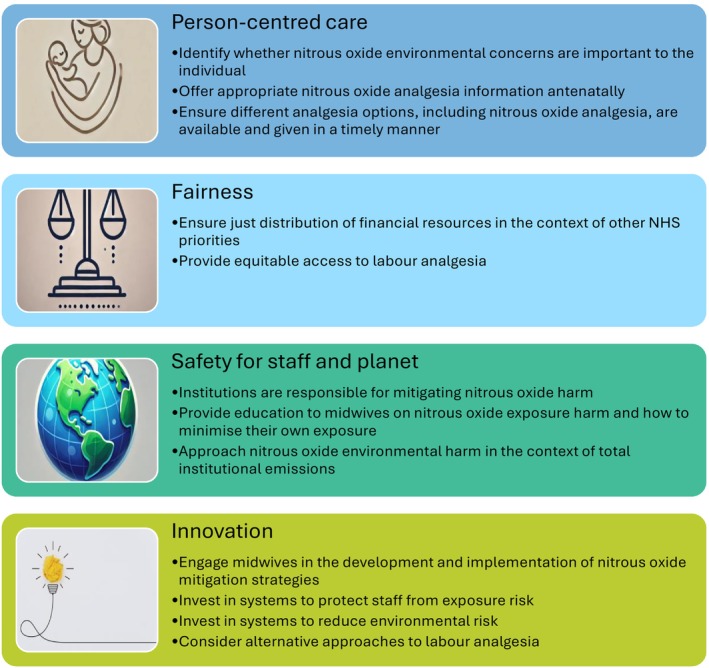
Framework for improvement includes considerations that should be made when implementing nitrous oxide mitigation strategies.

In this study, we interviewed people who had used nitrous oxide previously for labour analgesia to evaluate their perceptions of the environmental and occupational hazards of this intervention and explored the acceptability of different strategies aimed at reducing nitrous oxide emissions. We considered our study findings in the context of recommendations that have been made to mitigate the harmful impacts of nitrous oxide [10, 12, 13, 14, 15, 16] whilst maintaining parturient autonomy [21, 22], and have created a framework for consideration while recommendations continue to be developed and implemented (Fig. 1).

Although studies have evaluated the views of midwives and other healthcare workers on the environmental and occupational risks of nitrous oxide, we believe this to be the first study which has interviewed individuals with a recent experience of labour. Our findings broadly support those of a survey by The Health Foundation, which found that although the public generally supports the NHS Net Zero ambition, this is not as high a priority as other areas, such as “*hiring additional staff*” [19]. The Health Foundation also found that whilst some interventions, such as investing in greener vehicles, were backed by the public, measures that could directly impact their individual treatment decisions, such as considering environmental impact when deciding which treatments the NHS should offer, were less popular [19]. This reflects the views of participants who “*…do care about the environment and things like that, but for me, I think I do my bit elsewhere…so I wouldn't really care about the environment effects [of nitrous oxide]*” (Participant 5).

Nitrous oxide has been used for labour analgesia since the 1960s [32] and participants reported its use as being widespread (both in discussion and availability) across maternity culture. Its prevalence in the UK may reinforce the expectation, described by many of our interview participants, that it will be there for them to use. Current National Institute for Health and Care Excellence guidance recommends that nitrous oxide should be available in all birth settings [3]. The relative safety of nitrous oxide is reinforced by professional guidance, for example the public information website of the Obstetric Anaesthetists' Association states that “*Entonox^®^ has been used safely for pain relief for labour for many years*” but does not mention safety when describing other forms of pharmacological labour analgesia [33]. As articulated by one interviewee, the “*rhetoric is that it is safe*” (Participant 3).

Some of our study participants used nitrous oxide for labour analgesia despite not intending to do so or continued to use it despite finding it ineffective. The variable analgesic efficacy of nitrous oxide is well‐recognised [17], and the many non‐analgesic benefits of nitrous oxide were reported by our participants as being one of its advantages over other methods of labour analgesia. This supports existing research which has found that there are many complex contributors to maternal satisfaction with labour [34, 35, 36], including effective communication and being involved in decision‐making [37]. This supports the need to ensure that people giving birth have the option to access the full range of pain relief choices during labour, of which nitrous oxide is the one used most widely in the UK.

It is true that other health systems are lower users of nitrous oxide for labour analgesia, for example, the USA and parts of Europe [4]; furthermore, it was seen as a “*bonus*” by one of our participants who was from France. However, ensuring that people giving birth are safe and have as good an experience of childbirth as possible are key considerations when evaluating potential changes in practice or system design aimed at reducing the environmental impact of nitrous oxide. Delays in access to epidural analgesia were reported by some participants, and lack of pain relief was one of the key themes to emerge from the recent report by the All‐Party Parliamentary Group on Birth Trauma [38]. Alternatives to nitrous oxide, such as remifentanil patient‐controlled analgesia, have variable availability in the UK [39] and this option was not offered to any of our study participants. Socio‐economic deprivation is associated with lower utilisation of epidural analgesia, and reducing access to nitrous oxide may widen health inequalities [40]. Participants reported a concern about the care of people giving birth not being prioritised more generally.

Nitrous oxide use for labour analgesia may be reduced if people in labour choose to use it less, despite it still being available. Gynther et al. proposed that giving information about the carbon footprint of nitrous oxide supports a person's autonomy in making informed decisions about their labour and that “*this knowledge, coupled with the lack of good evidence for nitrous oxide's analgesic efficacy, may reduce the number of women choosing to use it for labour*” [20]. Our study participants were broadly in favour of environmental information being given but were unsure whether knowledge of the environmental impact of nitrous oxide would affect their choice of labour analgesia.

Overall, our participants did not feel it was appropriate to discuss the occupational exposure risks of nitrous oxide antenatally, even though they expressed concern about them. This is because they considered the protection of staff from occupational exposure to be a hospital responsibility. Guidance from the NHS is available to support Trusts on how to protect maternity staff by measuring and limiting their exposure to nitrous oxide [10]. There may also be scope for improved education of healthcare staff. Fewer than 10% of midwives in a recent survey had received any education on the environmental or occupational effects of nitrous oxide [41]. By contrast, these risks are highlighted in sedation guidance for dentists and dental nurses [42].

The effectiveness of current exhaled gas ‘cracking’ technology depends on equipment and technique, with up to 81% reduction in ambient nitrous oxide levels [15]. Although participants were supportive of using such technology (or other mitigation strategies) during labour, they questioned whether financial investment in reducing NHS emissions should be prioritised over other areas. Some reported that the occupational exposure benefits of any mitigating technologies add weight to the argument for investment because “*the two [emissions and occupational exposure] together feel like quite [an] immediate concern*” (Participant 3).

The strength of our study is that the qualitative methodology provided a rich, in‐depth understanding of the data. Our inductive approach allowed us to develop emergent themes that have not been reported previously. However, there are some limitations to this study. The subject and the way it was advertised may have attracted participants who are more environmentally conscious than most and would therefore be more interested to learn about and be influenced by the environmental effects of nitrous oxide. We only interviewed previous users of nitrous oxide for labour analgesia so we could explore their experience of this intervention; however, in doing so, we have not considered the views of those who have given birth without nitrous oxide. It is therefore possible that we excluded people who chose not to use nitrous oxide for environmental or occupational exposure concerns. The study was advertised through physical and virtual posters in community‐based parental groups, free community playgroups and parental social media networks in a particular geographical area (North London). The advert was only publicised in English, thus excluding those who do not read English. People who had a poor experience of childbirth (and potentially a poor experience of using nitrous oxide) may have been reluctant to be interviewed about their labour experience. We may have therefore recruited participants with disproportionately positive experiences of using nitrous oxide, although some of our participants did state they were not satisfied with it for their labour analgesia. Initial communications were done via email, so we could only recruit people with email addresses. We did not explore in depth the views of people who have used nitrous oxide for analgesia in other situations, such as for injuries or minor procedures. We conducted a relatively small number of interviews, but, as discussed above, we used an information power approach to determine sample size. Items of this study that point towards a larger sample size being required are our cross‐case analysis and the limited theoretical background since, to our knowledge, the views of people who have given birth on these topics have not been specifically researched before. Malterud et al. suggest that “*for an exploratory study, we do not head for a complete description of all aspects of the phenomenon we study. We are usually satisfied when a study offers new insights that contribute substantially to or challenge current understandings*” [25]. Finally, as authors, our perspectives and experiences may have influenced our analysis and interpretations. We have tried to address these through transparency and reflexivity, recognising that subjectivity is itself a resource in reflexive thematic analysis [43].

In conclusion, this study provides insights of the environmental and occupational exposure risks of nitrous oxide from the perspectives of people who have given birth using it for labour analgesia. Our findings can be used to support a person‐centred approach to reduce environmental harm. Overall responsibility for reducing environmental harm is at the institutional level; however, the specific context of antenatal care may provide a useful place to further explore how much patients want to understand the environmental impact of their care, and how much they may want to influence how healthcare systems respond to the climate crisis.

## Supporting information


**Appendix S1.** Recruitment poster text for members of the public.

**Appendix S2.** Participant information sheet for members of the public.

**Appendix S3.** Interview guide including main questions and supplementary probe questions.

Plain Language Summary
