# Supplementary material for: Views of people who have given birth on the environmental and occupational exposure risks of nitrous oxide for labour analgesia: an interview‐based qualitative study
Source: Anaesthesia. 2025 Jul 29;80(11):1333–42. doi: 10.1111/anae.16687 (PMC12519921; doi:10.1111/anae.16687)
Supplement: Supplementary file 1 — Appendix S1. Recruitment poster text for members of the public. [file ANAE-80-1333-s003.docx]

**Appendix S1** Recruitment poster text for members of the public

**Have you given birth in the last year?** **Or have you had a minor injury or procedure in the last year where you were offered ‘gas and air’ (or Entonox)?**

‘Gas and air’ (Entonox / nitrous oxide) is a medical gas frequently used pain relief that is very safe, short-acting and causes no long-term side effects when used appropriately. There are some concerns regarding its effects on the environment and on healthcare staff who are exposed to it for prolonged periods.

This project aims to explore the understanding and opinions of people who have used or been offered Entonox for pain relief regarding its effects on the environment and its potential effects on healthcare staff who are exposed to it for prolonged periods.

We are a team of researchers from UCL .

**We would love to hear from you.**

We are looking for people who used or considered nitrous oxide, otherwise known as “Gas and Air” or Entonox for pain relief during childbirth, during an endoscopy (camera test), or for a minor injury or procedure.

Please email *** if you are interested in being interviewed for this research. The interview will take 20 minutes and can be done in person or online at a time that is convenient to you. You will receive a £15 voucher as a thank you for your involvement.

This research is reviewed and approved by UCL Research Ethics Committee (Approval ID Number: 24905/001)
