# Supplementary material for: Views of people who have given birth on the environmental and occupational exposure risks of nitrous oxide for labour analgesia: an interview‐based qualitative study
Source: Anaesthesia. 2025 Jul 29;80(11):1333–42. doi: 10.1111/anae.16687 (PMC12519921; doi:10.1111/anae.16687)
Supplement: Supplementary file 2 — Appendix S2. Participant information sheet for members of the public. [file ANAE-80-1333-s004.docx]

**Appendix S2** Participant Information Sheet For Members of the Public

UCL Research Ethics Committee Approval ID Number: 24905/001

**YOU WILL BE GIVEN A COPY OF THIS INFORMATION SHEET**

**Title of Study: Use of nitrous oxide for analgesia and procedural sedation: interview study**

**Department: Department of Targeted Intervention**

**Name and Contact Details of the Researchers:**

Dr Tom Salih – Tomsalih@nhs.net

Dr Laura Elgie – L.elgie@nhs.net

**Name and Contact Details of the Principal Researcher:**

Prof Ramani Moonesinghe – Ramani.moonesinghe@nhs.net

1. **Invitation Paragraph**

You are being invited to take part in a research project.  Before you decide whether you would like to participate, it is important you understand why the research is being done and what participation will involve.  Please take time to read the following information carefully and discuss it with others if you wish. Please ask us if anything is not clear or you would like more information.  Thank you for your time and for considering participating in this research.

1. **What is the project’s purpose?**

The NHS is aiming to be the world’s first net zero carbon health service.  It is aiming for the emissions it controls directly to be net zero by 2040 and the emissions it influences to be net zero by 2045 ([NHS England, 2020](https://www.england.nhs.uk/greenernhs/wp-content/uploads/sites/51/2022/07/B1728-delivering-a-net-zero-nhs-july-2022.pdf)).  The carbon footprint of the NHS currently accounts for around 5% of the carbon footprint of the UK and reducing it needs, amongst other things, research into new technologies and equipment that can minimise emissions.  Medicines account for 25% of NHS emissions.  This project will explore the views of women who have used or been offered one particular medicine, Entonox, for childbirth, and patients who have received it for medical procedures.

Nitrous oxide is a medical gas frequently pain relief.  Entonox (also known as “gas and air”) is a mixture that is half nitrous oxide/half oxygen and is the form of nitrous oxide that is commonly used for pain relief in childbirth, for minor injuries and for some medical procedures.  It is a self-administered gas people that breathe in through a mouthpiece when they need it.  It has many advantages for the user: it is very safe, short-acting and causes no long-term side effects when used appropriately.  There are some concerns regarding its effects on the environment and on healthcare staff who are exposed to it for prolonged periods.

This **project aims to explore the understanding and opinions of** **people** who have used or been offered Entonox for pain relief regarding:

(a) its effects on the environment

(b) its potential effects on healthcare staff who are exposed to it for prolonged periods

(c ) research and technologies to reduce the above

This will be done either in person, on Microsoft Teams or by telephone interviews, that will last approximately 20 minutes.  The project will run from 01 April 2023 to 01 October 2023, but your direct involvement will only be for the duration of the interview.

We would like to emphasise that **we do not intend to dissuade anyone from using Entonox** and **believe it should be freely available** for people to choose to use as their pain relief.

1. **Why have I been chosen?**

You have been sent this information sheet because you may have used or been offered Entonox as a form of pain relief, or may have used a different form of pain relief either for childbirth or for some other medical reason.

1. **Do I have to take part?**

No.  Taking part in this study is entirely voluntary.  If you decide to take part you will be given this information sheet to keep and be asked to sign a consent form.  You can withdraw your consent and stop participating in the interview at any time, without giving a reason.  There will be no penalties for doing so.  If you decide to withdraw from the study after being interviewed we will destroy the data we have collected during your interview, as long as we have been notified before the data have been summarised for analysis.  *You will be able to withdraw your data up to 4 weeks after your interview.*

1. **What will happen to me if I take part?**

You will be approached by a researcher by email or telephone, inviting you to take part in an interview.  We will provide you with the participant information sheet and consent form at least 48 hours before the interview, so you have time to read it and ask any questions ahead of the interview.  Once you are happy with the information you have been given and any answers to your questions, we will ask you to return a signed copy of the consent form to us.  The interview will be voluntary, conducted either in person, on Microsoft Teams or by telephone call and last approximately 20 minutes.

During the interview, we will ask you if you have ever used Entonox and your experience of it.  If you used Entonox for pain relief in childbirth we will ask you to tell us about yourself and your delivery, including when and how your baby was born and the effectiveness and satisfaction you had with your pain relief.

We will also ask you about your awareness of the environmental and potential staff exposure effects of Entonox, and whether you think it is appropriate for these to be discussed with pregnant women.  We will ask you if you would consider the environmental effects of Entonox when choosing your pain relief and whether you would consider using new technologies to reduce these effects.  We will ask you if you think it is worthwhile investing in such new technologies.

All data we collect will be stored on (encrypted) UCL systems. Recordings will be deleted once transcription is complete.  Consent forms will be deleted at the end of the project.  Contact details will be deleted at the end of the project unless participants have consented to be contacted in future.

1. **Will I be recorded and how will the recorded media be used?**

We will take notes during the interview and an audio-recording will also be made using a digital recorder or the Microsoft Teams platform’s own recording software. Recorded interviews will be transcribed (written-up) and the recording will then be deleted.  The information we gather will be treated as confidential by the researchers and pseudonymised or anonymised for analysis.

1. **What are the possible disadvantages and risks of taking part?**

You may find the interview uncomfortable or upsetting as you recall painful events.  Please remember that you can withdraw from the interview at any point.  If this occurs, you will be directed to support services through your GP, midwife or health visitor.  The researchers are qualified doctors with experience discussing sensitive events.

Some parents feel upset, distressed or traumatised by childbirth and support is available.  We recommend the resources available on the Tommy’s PregnancyHub website for further information.

[www.tommys.org/pregnancy-information/after-birth/recovering-difficult-birth](http://www.tommys.org/pregnancy-information/after-birth/recovering-difficult-birth)

All the information you provide will be documented and anonymised, so your name will not be included in any reports or publications of this research.  If we have taken your phone number or email address for the purpose of conducting the interview, we will store these in a password protected file and delete them after the interview.

1. **What are the possible benefits of taking part?**

We hope that this work will help us understand if and how to discuss certain issues around Entonox with those who might use it for pain relief in a healthcare environment.  We hope that it will also contribute to the development of technologies that will mitigate against the environmental and repetitive exposure effects of Entonox. We will offer participants a payment voucher of £15 as a thank you for their time.

1. **What if something goes wrong?**

If you would like to raise a complaint please contact Professor Ramani Moonesinghe (contact details above).  If you feel that your complaint has not been handled to your satisfaction, you are welcome to contact the Chair of the UCL Research Ethics Committee ([ethics@ucl.ac.uk](mailto:ethics@ucl.ac.uk)).

1. **Will my taking part in this project be kept confidential?**

We are asking participants to supply contact details (name, email address and telephone number) so that they can be contacted for interview.  This information will be stored separately from the interview content so that the information you give in the interview cannot be identified to a particular participant.

All the information that we collect about you during the course of the research will be kept strictly confidential. You will not be able to be identified in any ensuing reports or publications.  During the interviews you will not be asked to provide any confidential information.  Any personal information that is given during interviews (e.g. names) will be removed from the interview transcripts.

1. **Limits to confidentiality**

Please note that confidentiality will be maintained as far as it is possible, unless during our conversation I hear anything which makes me worried that someone might be in danger of harm, I might have to inform relevant agencies of this.

1. **What will happen to the results of the research project?**

Audio and video recordings obtained during the interviews will be deleted after they have been transcribed and before the end of the project.  Transcripts will be retained in anonymised form with participant consent. The results of the research may contribute to publications within 2 years of the study completion, participants will not be identified in any report or publication.  Data will be used to inform strategies for nitrous oxide mitigation and may be used for additional or future research.

1. **Local Data Protection Privacy Notice**

**Notice:**

The controller for this project will be University College London (UCL). The UCL Data Protection Officer provides oversight of UCL activities involving the processing of personal data, and can be contacted at [data-protection@ucl.ac.uk](mailto:data-protection@ucl.ac.uk)

This ‘local’ privacy notice sets out the information that applies to this particular study. Further information on how UCL uses participant information can be found in our ‘general’ privacy notice: For participants in health and care research studies, click [here](http://www.ucl.ac.uk/legal-services/privacy/participants-health-and-care-research-privacy-notice)

The information that is required to be provided to participants under data protection legislation (GDPR and DPA 2018) is provided across both the ‘local’ and ‘general’ privacy notices.

The categories of personal data used will be as follows:

Name on the study informed consent form; phone number; email address

The lawful basis that would be used to process your *personal data* will be performance of a task in the public interest.

*Your personal data will be processed so long as it is required for the research project*. If we are able to anonymise or pseudonymise the personal data you provide we will undertake this and will endeavour to minimise the processing of personal data wherever possible.

If you are concerned about how your personal data is being processed, or if you would like to contact us about your rights, please contact UCL in the first instance at [data-protection@ucl.ac.uk](mailto:data-protection@ucl.ac.uk).

1. **Who is organising and funding the research?**

This research is organised by a research team from UCL.  The research is funded by the Small Business Research Initiative (SBRI)

1. **Contact for further information**

If you have any questions or would like to discuss thing further please contact:

Dr Tom Salih – [Tomsalih@nhs.net](mailto:Tomsalih@nhs.net)  Dr Laura Elgie – [L.elgie@nhs.net](mailto:L.elgie@nhs.net)

You will be given a copy of the information sheet and a signed consent form to keep.

**Thank you for reading this information sheet and for considering taking part in this research study.**
