# Supplementary material for: Views of people who have given birth on the environmental and occupational exposure risks of nitrous oxide for labour analgesia: an interview‐based qualitative study
Source: Anaesthesia. 2025 Jul 29;80(11):1333–42. doi: 10.1111/anae.16687 (PMC12519921; doi:10.1111/anae.16687)
Supplement: Supplementary file 3 — Appendix S3. Interview guide including main questions and supplementary probe questions. [file ANAE-80-1333-s001.docx]

**Appendix S3** Interview guide including main questions and supplementary probe questions

1. **Please can you tell me a bit about yourself and your delivery:**
   1. How old is your baby?
   2. How was your baby born (vaginal/instrumental/LSCS)?
   3. Did you use gas and air for pain relief?
   4. Were you satisfied with gas and air as your pain relief?
2. As mentioned in the PIS, the NHS is aiming to be Net Zero and this required professionals to look at all areas and work out where it is possible to reduce our emissions. One of these areas is the medicine we use, and this project is focussing on gas and air in particular.

Gas and air is very safe for those who use it for childbirth or in other circumstances requiring pain relief, but there are some other concerns about it. We do not intend to dissuade anyone from using gas and air and believe it should be available as a choice for pain relief for childbirth and other conditions. We are interested to hear your views on these other concerns.

**Were you aware of the environmental effects of gas and air, prior to participating in this project?**

- 1. Where did you hear about these?
  2. Have you discussed them with anyone?
  3. Do you think it is reasonable for healthcare professionals to be discussing these effects with women, so they can make an informed decision?
  4. When do you think it would be best to discuss these effects?

1. **Would you factor in the environmental effects of gas and air when choosing your pain relief for childbirth?**
   1. Would you be interested in knowing the environmental effects of the different types of pain relief for childbirth?
2. Gas and air use during labour does not cause any long-term effects to mother and baby. It can, however, affect healthcare staff who are frequently exposed to it, so organisations have a duty to have systems in place to protect their staff from that risk.

**Were you aware of the potential effects of Entonox to staff who are frequently exposed to it, prior to participating in this study?**

- 1. Where did you hear about this?
  2. Have you discussed this with anyone?
  3. Do you think it is reasonable for healthcare professionals to be discussing these effects with women who are considering using gas and air, so they can make an informed decision?

1. **Would you be willing to use a new technology that would reduce the environmental impact of gas and air?**
   1. Is there anything that would make it easier for you to use this technology?
   2. Is there anything that would make it more difficult for you to use this technology?
2. **Do you think it is worth investing in technologies that reduce the environmental effects of gas and air?**
   1. Give info on cost of childbirth (e.g. antenatal care is £1,590 to £4,233. A straightforward birth with the shortest possible stay in hospital will cost around £3,282. Postnatal care can cost up to £1,207.50)
