# Supplementary material for: Views of people who have given birth on the environmental and occupational exposure risks of nitrous oxide for labour analgesia: an interview‐based qualitative study
Source: Anaesthesia. 2025 Jul 29;80(11):1333–42. doi: 10.1111/anae.16687 (PMC12519921; doi:10.1111/anae.16687)
Supplement: Supplementary file 4 — Plain Language Summary [file ANAE-80-1333-s002.docx]

**Plain Language Summary**

In the UK, many people use a gas called nitrous oxide mixed with oxygen to help with pain during childbirth. But nitrous oxide can harm the environment and may not be safe for hospital staff if not handled properly. To help protect the planet and reach ‘net zero’ (which means not adding more pollution than we take away), hospitals might need to change how they use this gas or find other ways to help with pain during labour. This study wanted to understand what people who used nitrous oxide during childbirth think, especially about how it affects the environment and hospital staff. We talked to 12 people who used nitrous oxide during labour. We asked them open-ended questions to hear their thoughts. Then we looked for patterns in what they said. We found three main ideas: nitrous oxide is the usual choice for pain relief during labour in the UK; people care about the environment, but what matters most depends on their personal situation; and people think hospitals, not mothers, should take care of making nitrous oxide safer for the environment and staff. Most people felt nitrous oxide was a safe and easy option, especially when they didn’t have many choices. They were curious to learn more about how it harms the environment but said this might not stop them from using it. They weren’t too worried about the risks to staff and thought hospitals should be the ones to fix that. While they supported helping the environment, they also thought the NHS might have more urgent needs for its money. This study gives helpful ideas for creating plans to reduce nitrous oxide pollution that also respect the needs and opinions of people who use it during childbirth.
